# Classification and risk stratification of invasive breast carcinomas using a real-time quantitative RT-PCR assay

**DOI:** 10.1186/bcr1399

**Published:** 2006-04-20

**Authors:** Laurent Perreard, Cheng Fan, John F Quackenbush, Michael Mullins, Nicholas P Gauthier, Edward Nelson, Mary Mone, Heidi Hansen, Saundra S Buys, Karen Rasmussen, Alejandra Ruiz Orrico, Donna Dreher, Rhonda Walters, Joel Parker, Zhiyuan Hu, Xiaping He, Juan P Palazzo, Olufunmilayo I Olopade, Aniko Szabo, Charles M Perou, Philip S Bernard

**Affiliations:** 1 The ARUP Institute for Clinical and Experimental Pathology, SLC, Utah, USA; 2 Department of Genetics and Department of Pathology & Laboratory Sciences, Lineberger Comprehensive Cancer Center, University of North Carolina, Chapel Hill, North Carolina, USA; 3 Department of Pathology, University of Utah School of Medicine, SLC, Utah, USA; 4 Department of Surgery, University of Utah School of Medicine, SLC, Utah, USA; 5 Department of Internal Medicine, University of Utah School of Medicine, SLC, Utah, USA; 6 Department of Clinical Genetics, Maine Center for Cancer Medicine, Scarborough, Maine, USA; 7 Department of Pathology, Thomas Jefferson University, Philadelphia, Pennsylvania, USA; 8 Constella Health Sciences, Durham, North Carolina, USA; 9 Department of Medicine, University of Chicago, Illinois, USA; 10 Department of Oncological Sciences, Huntsman Cancer Institute, SLC, Utah, USA

## Abstract

**Introduction:**

Predicting the clinical course of breast cancer is often difficult because it is a diverse disease comprised of many biological subtypes. Gene expression profiling by microarray analysis has identified breast cancer signatures that are important for prognosis and treatment. In the current article, we use microarray analysis and a real-time quantitative reverse-transcription (qRT)-PCR assay to risk-stratify breast cancers based on biological 'intrinsic' subtypes and proliferation.

**Methods:**

Gene sets were selected from microarray data to assess proliferation and to classify breast cancers into four different molecular subtypes, designated Luminal, Normal-like, HER2+/ER-, and Basal-like. One-hundred and twenty-three breast samples (117 invasive carcinomas, one fibroadenoma and five normal tissues) and three breast cancer cell lines were prospectively analyzed using a microarray (Agilent) and a qRT-PCR assay comprised of 53 genes. Biological subtypes were assigned from the microarray and qRT-PCR data by hierarchical clustering. A proliferation signature was used as a single meta-gene (log_2 _average of 14 genes) to predict outcome within the context of estrogen receptor status and biological 'intrinsic' subtype.

**Results:**

We found that the qRT-PCR assay could determine the intrinsic subtype (93% concordance with microarray-based assignments) and that the intrinsic subtypes were predictive of outcome. The proliferation meta-gene provided additional prognostic information for patients with the Luminal subtype (*P *= 0.0012), and for patients with estrogen receptor-positive tumors (*P *= 3.4 × 10^-6^). High proliferation in the Luminal subtype conferred a 19-fold relative risk of relapse (confidence interval = 95%) compared with Luminal tumors with low proliferation.

**Conclusion:**

A real-time qRT-PCR assay can recapitulate microarray classifications of breast cancer and can risk-stratify patients using the intrinsic subtype and proliferation. The proliferation meta-gene offers an objective and quantitative measurement for grade and adds significant prognostic information to the biological subtypes.

## Introduction

Current management of breast cancer is based on anatomic staging (tumor size/node involvement/metastasis) and morphological features such as the tumor grade [1]. Although anatomic staging and histological grade are important prognostic factors [2], they often fail to predict the clinical course of the disease. In order to improve upon the standard of care for breast cancer, there is a need for new molecular markers and diagnostic algorithms.

Microarray studies have shown that differences in gene expression can account for much of the diversity in breast cancer and that these profiles have prognostic significance [3-8]. A common method to find similarities (and differences) in the biology of breast cancer is to hierarchical cluster an 'intrinsic' gene set [3-5]. By definition, intrinsic genes have a large variation in expression across tumors from different individuals but have little variation in expression between biological replicates from the same individual; intrinsic genes therefore identify distinct tumor biology that could explain differences in phenotype (for example, drug response).

Hierarchical clustering of microarray data using an intrinsic gene set has shown that breast cancers can be classified into at least four groups: Luminal, Normal-like, HER2+/ER, and Basal-like [3]. Additional studies using larger numbers of patients have shown that these subtypes can be identified in independent data sets, and that the different classes are prognostic [5,6,9].

Breast tumors of the 'Luminal' subtype are estrogen receptor (ER)-positive and have a similar keratin expression profile to the epithelial cells lining the lumen of the breast ducts [4,5,10,11]. Conversely, ER-negative tumors can be divided into two main subtypes – namely those that overexpress (and are DNA amplified for) HER2 and GRB7 (HER2+/ER-), and 'Basal-like' tumors that have an expression profile similar to basal epithelium and express keratin 5, keratin 6B, keratin 14 and keratin 17. The ER-negative tumor subtypes are aggressive and typically more deadly than Luminal tumors; however, there are subtypes of Luminal tumors that lead to poor outcomes despite being ER-positive [3,4,6]. For example, Sorlie and colleagues identified a Luminal B subtype with similar outcomes to the HER2+/ER- and Basal-like subtypes [4], and Sotiriou and colleagues showed that there are three different types of Luminal tumors with different outcomes [6]. The Luminal tumors with poor outcomes consistently share the histopathological feature of being higher grade and the molecular feature of showing high expression of proliferation genes [4-6].

Proliferation genes are cell-cycle-regulated genes that have a variety of functions necessary for cell growth, DNA replication, and mitosis [12,13]. Despite their diverse functions, proliferation genes have similar gene expression profiles when analyzed by hierarchical clustering. Furthermore, studies using supervised analyses to find genes that predict outcome commonly identify proliferation genes. For example, the SAM264 'survival' list stated in Sorlie and colleagues [4], the 231 'prognosis classifier' list of van 't Veer and colleagues [7], and the 485 prognostic genes presented in Sotiriou and colleagues [6] all contained proliferation genes, suggesting that all of these studies are probably tracking a similar phenotype.

The main objectives of this study are to compare molecular subtype classification between microarray analysis and real-time quantitative reverse-transcription (qRT)-PCR analysis, and to assess the prognostic significance of a proliferation meta-gene, both as an independent marker and within the context of the breast cancer subtypes.

## Materials and methods

### Patient selection

An ethnically diverse cohort of patients was studied using samples collected from the University of Utah Health Sciences Center, from the University of North Carolina, from Thomas Jefferson University, from the Maine Medical Center, and from the University of Chicago. Patients provided written acknowledgement of informed consent in accordance with institutional and federal guidelines. Samples collected prospectively for microarray and qRT-PCR analyses included 117 invasive breast cancers, one fibroadenoma, five 'normal' samples (from reduction mammoplasty), and three cell lines. Patients were treated in accordance with the standard of care dictated by their disease stage, ER status, and HER2 status. Patient outcome information was collected for up to 118 months (median 21.5 months). The clinical data for the qRT-PCR samples are presented in Additional file 2 (Supplemental Table 1). Publicly available data sets containing 337 samples with long-term follow-up (median 86.7 months) were used to further validate the prognostic significance of the proliferation meta-gene within the context of intrinsic subtypes [7,8,14].

### Sample preparation and first-strand synthesis for qRT-PCR

Nucleic acids were extracted from fresh frozen tissue using the RNeasy Midi Kit (Qiagen Inc., Valencia, CA, USA). The quality of RNA was assessed using the Agilent 2100 Bioanalyzer with the RNA 6000 Nano LabChip Kit (Agilent Technologies, Palo Alto, CA, USA). All samples used had discernable 18S and 28S ribosomal peaks. First-strand cDNA was synthesized from approximately 1.5 μg total RNA using 500 ng Oligo(dT)_12–18 _and Superscript III reverse transcriptase (1^st ^Strand Kit; Invitrogen, Carlsbad, CA, USA). The reaction was held at 42°C for 50 minutes followed by a 15-minute step at 70°C. The cDNA was washed on a QIAquick PCR purification column and was stored at -80°C in 25 mM Tris, 1 mM ethylenediamine tetraacetic acid at a concentration of 5 ng/μl (concentration estimated from the starting RNA concentration used in the reverse transcription).

### Primer design

Genbank sequences were downloaded from Evidence viewer (NCBI website) into the Lightcycler Probe Design Software (Roche Applied Science, Indianapolis, IN, USA). All primer sets were designed to have a Tm of approximately 60°C, to have a GC content of approximately 50%, and to generate a PCR amplicon <200 bps. Finally, BLAT and BLAST searches were performed on primer pair sequences using the UCSC Genome Bioinformatics database and the NCBI database to check for uniqueness. Primer sets and identifiers are provided in Additional file 2 (Supplemental Table 2).

### Real-time PCR

For PCR each 20 μl reaction included 1 × PCR buffer with 3 mM MgCl_2 _(Idaho Technology Inc., Salt Lake City, UT, USA), 0.2 mM each of dATP, dCTP, and dGTP, 0.1 mM dTTP, 0.3 mM dUTP (Roche Applied Science), 10 ng cDNA and 1 U Platinum Taq (Invitrogen). The dsDNA dye SYBR Green I (Molecular Probes, Eugene, OR, USA) was used for all quantification (1/50,000 final). PCR amplifications were performed on the Lightcycler software using an initial denaturation step (94°C, 90 seconds) followed by 50 cycles: denaturation (94°C, 3 seconds), annealing (58°C, 5 seconds with 20°C/s transition), and extension (72°C, 6 seconds with 2°C/sec transition). Fluorescence (530 nm) from the dsDNA dye SYBR Green I was acquired for each cycle after the extension step. The specificity of the PCR was determined by postamplification melting curve analysis. Reactions were automatically cooled to 60°C at a rate of 3°C/s and slowly heated at 0.1°C/s to 95°C while continuously monitoring the fluorescence.

### Relative quantification by real-time qRT-PCR

Quantification was performed using the LightCycler 4.0 software. The crossing threshold for each reaction was determined using the second-derivative maximum method [15,16]. The relative copy number was calculated using an external calibration curve to correct for PCR efficiency and a within-run calibrator to correct for the variability between runs. The calibrator is made from four equal parts of RNA from three cell lines (MCF7, SKBR3, and ME16C) and Universal Human Reference RNA (catalogue number #740000; Stratagene, La Jolla, CA, USA).

Differences in cDNA input were corrected by dividing the target copy number by the arithmetic mean of the copy number for three housekeeper genes (MRPL19, PSMC4, and PUM1) [17]. After adjusting copy numbers to the reference sample (calibrator) in LCS4, relative copy numbers were imported into a relational database where the data were normalized to the housekeeper genes and were log_2_-transformed for further analyses.

Hierarchical clustering was carried out in Cluster analysis using Spearman correlation, median centering by gene and array, and average linkage association [18]. The clustering was visualized using Treeview. The real-time qRT-PCR relative copy number data for all genes (53 classifier genes and three housekeeper genes) can be found in Additional file 2 (Supplemental Table 3).

### Histopathology/immunohistochemistry

Histological assessment of grade was performed for the invasive ductal adenocarcinomas using the Scarff-Bloom-Richardson system. Nuclear grading was determined for tumors in which tubular differentiation could not be assessed (for example, invasive lobular carcinomas). Samples were scored for protein expression at the time of diagnosis and using standard operating procedures established at each institution. Greater than 20% positive staining nuclei was considered positive for the ER and the progesterone receptor. Staining and scoring criteria for HER2 were carried out according to the HercepTest (Dako, Carpinteria, CA, USA).

### Microarray experiments

The 126 samples used for qRT-PCR were also analyzed by DNA microarray (Agilent Human A1, Agilent Human A2, and custom oligonucleotide). Labeling and hybridization of RNA for microarray analysis were performed using the Agilent low RNA input linear amplification kit, but with one-half of the recommended reagent volumes and using a Qiagen PCR purification kit to clean up the cRNA. Each sample was assayed versus a common reference sample that was a mixture of Human Universal Reference total RNA (Stratagene, La Jolla, CA, USA) enriched with equal amounts of RNA from the MCF7 and ME16C cell lines. Microarray hybridizations were carried out on Agilent Human oligonucleotide microarrays using 2 μg Cy3-labeled 'reference' sample and 2 μg Cy5-labeled 'experimental' sample. Hybridizations were carried out using the Agilent hybridization kit and a Robbins Scientific '22k chamber' hybridization oven (Robbins Scientific, Sunnyvale, CA, USA). The arrays were incubated overnight, washed once in 2 × SSC and 0.0005% Triton X-102 (10 minutes), washed twice in 0.1 × SSC (5 minutes), and were then immersed into Agilent Stabilization and Drying solution for 20 seconds.

All microarrays were scanned using an Axon Scanner 4000A (Axon Instruments, Inc, Foster City, CA, USA). The image files were analyzed with GenePix Pro 4.1 (Axon Instruments) and were uploaded into the UNC Microarray Database at the University of North Carolina at Chapel Hill, where a lowest normalization procedure was performed to adjust the Cy3 and Cy5 channels [19]. All primary microarray data associated with this study are available at the UNC Microarray Database and have been deposited in the GEO under accession number GSE2607.

### Selecting genes for real-time qRT-PCR

We developed a real-time qRT-PCR assay using 53 genes that were selected due to their importance in making 'intrinsic' subtype distinctions and/or their association with cell proliferation (see Additional file 2, Supplemental Table 2). The statistical selection of 'intrinsic' genes involved using 45 before-therapy and after-therapy samples derived from the data set presented in Sorlie and colleagues (see Additional file 2, Supplemental Table 4 for the list of 45 pairs) [5]. The two-color DNA microarray data were downloaded from the Internet and the R/G ratio (experimental/reference) for each spot was normalized and log_2_-transformed. Missing values were imputed using the k-NN imputation algorithm described by Troyanskaya and colleagues [20].

Using an 'intrinsic' analysis [3] we identified 550 microarray elements/spots from the data set presented by Sorlie and colleagues [5]. We then applied the 'intrinsic' genes to identifying molecular subtypes within a completely independent data set of early-stage breast cancers [7]. Common elements between the data sets were found after translating the gene annotation from each data set to UniGene Cluster IDs using the SOURCE database [21]. Following the algorithm outlined by Tibshirani and colleagues [22,23], we hierarchical clustered the 97 samples from van 't Veer and colleagues' study [7] using a common set of 350 genes and assigned intrinsic subtypes (Luminal, HER2+/ER-, Basal-like, or Normal-like) based on the sample-associated dendrogram. Finally, we identified genes that optimally distinguished the four subtype classes using a version of the gene selection method first described by Dudoit and Fridlyand [24], where the best class distinguishers are identified according to the ratio of between-group to within-group sums of squares. After scoring genes in this manner, 10-fold cross-validation was performed with a nearest centroid classifier, resulting in a list of 41 genes that gave the highest prediction accuracy when compared with the entire set of 350 genes.

We successfully developed qRT-PCR assays for 37 out of the 41 genes identified from the 'intrinsic' analysis (minimal intrinsic list). The genes PGR and EGFR were also included in the qRT-PCR assay, despite not being statistically selected in the 'intrinsic' analysis, because of their value in predicting therapy response and their strong association with ER-positive and ER-negative tumors. Finally, we tested 14 proliferation genes because of their importance in prognosis.

### Cluster robustness

The stability of our hierarchical clustering classifications was tested using a *k*-means algorithm implemented in Cluster 3.0 and using Consensus Cluster [25] implemented in GenePattern. For the *k*-means we ran 1,000 trials (*K *= 4 and *K *= 5) and used the Euclidean distance as the similarity metric. Consensus clustering was also performed for 1,000 runs using a 'subsampling' of 0.8, so that 20% of the samples were left out of each run.

### Combining microarray and qRT-PCR datasets

We used distance-weighted discrimination (DWD) to identify and correct systematic biases across the microarray and qRT-PCR datasets [26]. Prior to DWD, we normalized each dataset by setting the mean to 0 and the variance to 1. After performing DWD, genes in common between the datasets were clustered using Spearman correlation and average linkage association.

### Receiver operator curves

In order to determine agreement between protein expression (immunohistochemistry (IHC)) and gene expression (qRT-PCR), a cutoff value for the relative gene copy number was selected by minimizing the sum of the observed false-positive and false-negative errors; that is, minimizing the estimated overall error rate under equal priors for the presence/absence of the protein. The sensitivity and specificity of the resulting classification rule were estimated via bootstrap adjustment for optimism [27].

### Survival analyses

Survival curves were estimated by the Kaplan-Meier method and compared via a log-rank or stratified log-rank test as appropriate. The standard clinical pathological parameters of age (years), node status (positive versus negative), tumor size (cm, a continuous variable), grade (1–3, a continuous covariate), and ER status (positive versus negative) were tested for differences in relapse-free survival (RFS) and overall survival using the Cox proportional hazards regression model. Pairwise log-rank tests were used to test for equality of the hazard functions among the intrinsic classes. Cox regression was used to determine predictors of survival from continuous expression data. All statistical analyses were performed using the R statistical software package (R Foundation for Statistical Computing Vienna, Austria).

## Results and discussion

### Recapitulating microarray-based breast cancer classifications using qRT-PCR

Two major challenges in using genomics for breast cancer diagnostics are the ability to find robust classifications that maintain prognostic significance across different patient populations, and the ability to effectively translate those classifications into the clinical laboratory. Microarray studies on breast cancer have shown that particular signatures, such as those for intrinsic subtype classification and proliferation, are consistently identified and are prognostic across different data sets [3-5,7,8,14,28]. In order to determine whether the 'intrinsic' classifications found by microarray analysis could be generated from real-time qRT-PCR data, we prospectively compared the two platforms by profiling 126 different breast tissue samples (117 invasive, five normal, one fibroadenoma, and three cell lines) with Agilent microarrays (20,000 elements) and a real-time qRT-PCR assay. The qRT-PCR assay was comprised of 53 genes that were selected to optimally identify the four main breast tumor intrinsic subtypes [3,4], and to create an objective gene expression predictor for cell proliferation and outcome [12,29,30].

A major objection to using hierarchical clustering for defining tumor subtypes is that the algorithms are designed for associating and visualizing gene expression patterns, and are not necessarily designed for sample classification. We therefore tested the 'robustness' of the hierarchical clustering classifications using a *k*-means method and consensus clustering [25]; both methods found that there were at least four stable clusters.

**Figure 1:**
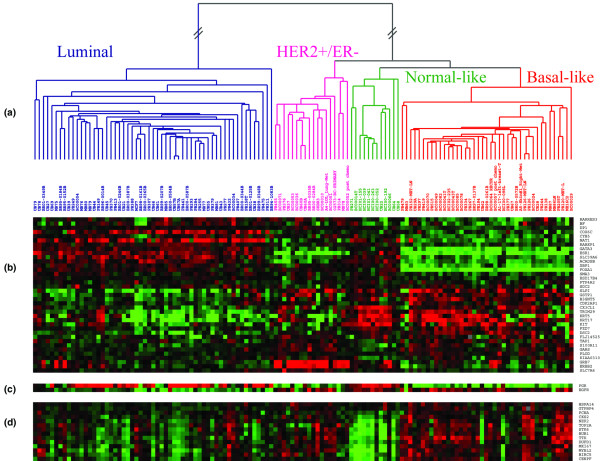
Two-way hierarchical clustering of real-time quantitative reverse-transcription (qRT)-PCR data. **(a) **The sample-associated dendrogram groups the 126 breast samples profiled by qRT-PCR into the same classes seen by microarray analysis. Samples are grouped into Luminal (blue), HER2+/ER- (pink), Normal-like (green), and Basal-like (red) subtypes. The expression level for each gene is shown relative to the median expression of that gene across all the samples, with high expression represented by red and low expression represented by green. Genes with median expression are black and missing values are gray. **(b) **A minimal set of 37 'intrinsic' genes was used to classify tumors into their primary 'intrinsic' subtypes. The 'intrinsic' gene set was supplemented using **(c) **PgR and EGFR, and **(d) **proliferation genes. The genes in (c) and (d) were clustered separately in order to determine agreement between the minimal 37 qRT-PCR 'intrinsic' set and the larger 402 microarray 'intrinsic' set (see Additional file 1, Supplemental Figure 1). Overall, 114/123 (93%) primary breast samples were classified the same between microarray and qRT-PCR.

There were 402 genes in common between our current microarray data set and the 550 intrinsic genes initially identified using the 45 paired samples taken from Sorlie and colleagues [5]. Two-way hierarchical clustering of the same 126 samples using either microarray data for the 402 'intrinsic' genes (Additional file 1, Supplemental Figure 1) or qRT-PCR data for the minimal 37 'intrinsic' genes (Figure 1) showed 93% concordance in classification. The samples were grouped into Luminal, HER2+/ER-, Normal-like, and Basal-like subtypes by both platforms.

In the qRT-PCR classification, 49 out of 55 (89%) Luminal tumors with available IHC data were scored positive for ER. Conversely, 46 out of 54 (85%) tumors classified as HER2+/ER- or Basal-like were ER-negative by IHC. A 37-gene qRT-PCR assay using a minimal intrinsic list can therefore accurately identify intrinsic subtypes and make classifications that agree with the ER status.

### Comparing real-time qRT-PCR with immunohistochemistry

Current molecular classifications in surgical pathology are made by evaluating single markers rather than sets of markers. We therefore assessed single qRT-PCR markers for their sensitivity/specificity in determining IHC status (Additional file 1, Supplemental Figure 2) [31]. This was done for similar markers (for example, ESR1 gene expression compared with ER protein status) and for surrogate markers (for example, GATA3 gene expression compared with ER protein status). We found that the gene expression of ESR1 alone had 87% sensitivity and 90% specificity for predicting the ER status by IHC. The gene with the highest correlation in expression to ESR1 was GATA3 (0.79), and GATA3 alone showed 90% sensitivity and 81% specificity. In addition, gene expression of PgR correlated well with the progesterone receptor IHC status (sensitivity = 89%, specificity = 82%). There was high correlation in expression between HER2/ERBB2 and GRB7 (0.91), which are physically located near one another on chromosome 17q12 and are commonly overexpressed and DNA-amplified together. Both ERBB2 (sensitivity = 55%, specificity = 87%) and GRB7 (sensitivity = 40%, specificity = 96%) had low sensitivity but high specificity in predicting the HER2 status by IHC. It is not surprising that there was poor agreement between ERBB2 gene expression and HER2 scoring since IHC is known to overestimate HER2 status when compared to fluorescence in-situ hybridization [32].

### Proliferation and grade

Proliferation genes have a high correlation with grade and have been shown to be a major determinant of outcome in breast cancer, especially in predicting recurrence in ER-positive tumors and in women with early-stage disease [7,8,28,33,34]. For instance, proliferation is a predominant component of both the Oncotype Dx test (five out of 16 genes are proliferation markers) [33] and the MammaPrint^® ^microarray assay based on the 70-gene prognosis signature [7]. Several of the cell cycle genes identified (STK6, BUB1, and BIRC5) in those studies were also strong predictors of recurrence in this study and were part of our 14-gene proliferation signature.

Analysis of the real-time qRT-PCR data from our 14 selected 'proliferation' genes (Figure 1d) showed that Luminal tumors have relatively low replication activity compared with HER2+/ER- and Basal-like tumors. As expected, the Normal-like samples showed the lowest expression of the 'proliferation' genes. When correlating (Spearman correlation) the gene expression of all 53 qRT-PCR genes with grade, we found that the top genes with a positive correlation (for instance, high expression correlates with high grade) were the proliferation genes (Table 1). Since the significance of single markers may change depending on the cohort studied, we also created a proliferation meta-gene (log_2 _average of all 14 proliferation genes) as a potentially more robust measure of grade. The proliferation meta-gene was more highly correlated with grade (*r*_*s *_= 0.46) than any other single marker, except CENPF. Genes within the ER cluster (ESR1, GATA3, and XBP1) all had significant negative correlations with grade (Additional file 2, Supplemental Table 5).

Analysis of the proliferation cluster by gene ontology reveals that these coordinately expressed genes have diverse but complementary functions important for progression through the cell cycle, such as DNA replication (PCNA), chromosome segregation (TOP2A and STK6), and control of cell-cycle checkpoints (BUB1, MYBL2, and TTK). Although most of the proliferation genes are overexpressed in high-grade tumors regardless of their ER status, we found some genes (for example, NEK2A) that are 'good' proliferation markers for ER-negative tumors but not for ER-positive tumors. Genes functioning in cell polarity and adhesion were not represented in the proliferation genes, which is notable given that differentiation is an important aspect of histological grade. It is possible that genes important for tubule differentiation just do not cluster with cell-cycle-regulated genes or that these functions have yet to be revealed for genes in the proliferation cluster.

### Using qRT-PCR assay for predicting survival

**Figure 2:**
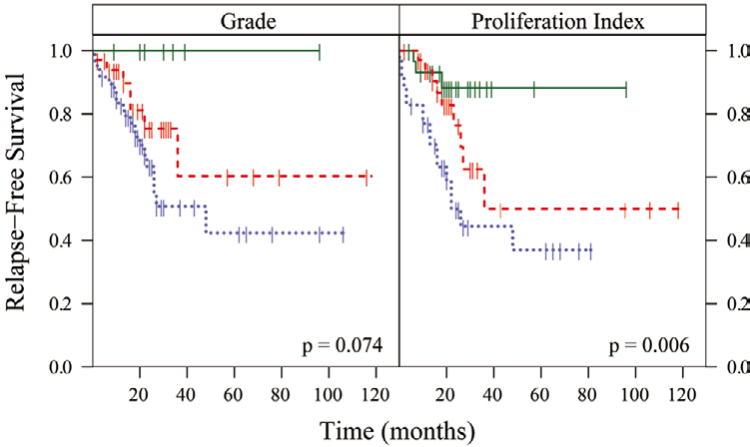
Grade and proliferation as predictors of relapse-free survival. A Cox regression model was used to determine probability of relapse over time. Kaplan-Meier curves show the time to event given different grades and levels of proliferation. The grade was scored as low (green), medium (red) or high (blue). The proliferation score was based on continuous expression data, and is shown as tertiles that correspond to low (green), medium (red), and high (blue) levels of expression. The proliferation meta-gene (log_2 _average of the 14 proliferation genes) showed significant value in predicting relapse, even after correcting for other clinical parameters important for survival (Table 1). Furthermore, when we include both the grade and proliferation in a model for relapse-free survival, we find that the proliferation meta-gene is the better predictor (grade, *P *= 0.51; proliferation index, *P *= 0.047).

Outcome analyses for the intrinsic subtypes showed that patients with Luminal tumors showed significantly better outcomes for RFS and overall survival compared with HER2+/ER- and Basal-like tumors (Additional file 1, Supplemental Figure 3). There was no difference in outcome between patients with HER2+/ER- and Basal-like tumors, with both groups doing poorly. In addition to determining the prognostic value of the biological 'intrinsic' subtypes, we also correlated individual 'intrinsic' classifiers (Additional file 2, Supplemental Table 5) and proliferation genes (Table 1) to the RFS and grade. We found that the proliferation meta-gene has significant predictive value for RFS (*P *= 0.003), even after adjusting for other important determinants of survival (Table 1 and Figure 2). Since lobular cancers can only be graded on nuclear contours and not on tubule differentiation, we also performed our analyses using ductal carcinomas only and found that proliferation was still a better predictor than grade (*P *= 0.022 versus *P *= 0.083). It should be noted that the proliferation signature was not evaluated in the Normal-like breast group because this group included control samples and few cancer samples.

**Figure 3:**
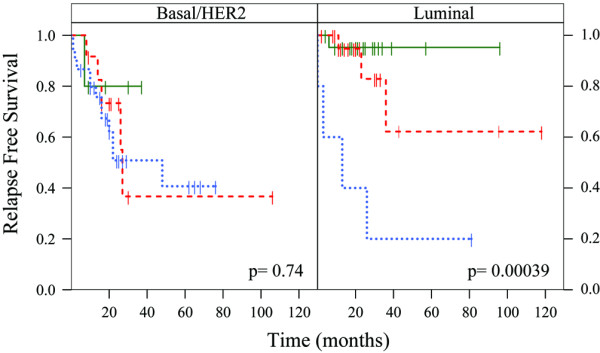
Intrinsic subtype stratified by the proliferation index. Tumors were given an 'intrinsic' subtype assignment based on the minimal 37-gene quantitative reverse-transcription-PCR classifier (Figure 1b). Patients were classified as having Luminal (estrogen receptor (ER)-positive) or HER2/Basal (ER-negative) subtypes. In order to have groups of similar size and because the subtypes largely follow ER status, tumors in the HER2 and Basal-like groups (both ER-negative) were combined. Continuous expression data for the proliferation meta-gene (log_2 _average of the 14 selected markers) were used in a Cox regression model to determine the probability of relapse over time. Differences in relapse for low (green), medium (red), and high (blue) expression are shown as tertiles in the Kaplan-Meier plots. Stratification by proliferation added information for relapse in the Luminal subtype (*P *= 0.00039) but not the ER-negative subtypes (*P *= 0.74).

Because the intrinsic subtypes (and ER status) capture much of the biology that explains variations in outcome among breast cancer patients, we tested whether the proliferation meta-gene added prognostic value to these classifications. When we separated tumors by intrinsic subtype (and ER status), and then stratified by the proliferation meta-gene, we found that proliferation only added prognostic information in the Luminal (and ER-positive) subtype of tumors (Figure 3). Women that had Luminal tumors with high proliferation were at a 19-fold increased risk of relapse compared with women that had Luminal tumors with low proliferation. Similarly, ER-positive tumors with high proliferation conferred a 13-fold relative risk of relapse.

Finally, we included the genomic classifiers (intrinsic subtype and proliferation) in multivariate survival analyses with standard clinical pathological information (Additional file 2, Supplemental Tables 6–9). Three multivariate models were applied to evaluate the contribution from standard clinical parameters alone (Model 1), from standard parameters plus genomic proliferation (Model 2), and from standard parameters plus proliferation and the intrinsic subtype (Model 3). This was performed for RFS (Additional file 2, Supplemental Tables 6 and 8) and for overall survival (Additional file 2, Supplemental Tables 7 and 9). The cohort analyzed by qRT-PCR showed that, without the addition of genomic classifiers, the top predictors for RFS were the tumor size, the node status, and the ER status. The proliferation meta-gene was a significant and independent predictor of survival in the multivariate analysis. The Luminal (ER-positive) versus Basal-like (ER-negative) distinction was significant for overall outcome in the multivariate analysis, even in the presence of standard ER status by IHC (Additional file 2, Supplemental Table 7).

### Testing the proliferation meta-gene for relapse in early-stage breast cancers

In order to further validate our observations and to determine whether our proliferation meta-gene was simply identifying the Luminal B subtype of tumors, previously described as having high proliferation and poor outcome [4], we applied the proliferation meta-gene to a large microarray dataset containing 337 patients with long-term follow-up and containing a Luminal B group. This microarray data set is the combined and nonredundant sample sets presented in van 't Veer and colleagues [7] and in Chang and colleagues [14], and represents a set of breast cancer patients from The Netherlands Cancer Institute (NKI dataset). Each sample was assigned an 'intrinsic subtype' using five subtype centroids (Luminal A, Luminal B, Basal-like, HER2+/ER-, and Normal-like), as described earlier.

**Figure 4:**
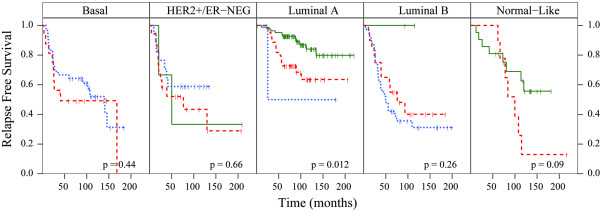
Stratification of five 'intrinsic' subtypes by the proliferation meta-gene. A large microarray breast cancer data set (337 samples × 16,000 genes) from women with early-stage disease was used to confirm the significance of the proliferation meta-gene to further risk-stratify the Luminal tumors. Tumors were classified as Basal, HER2+/ER-, Luminal A, Luminal B, and Normal-like. The microarray data for the proliferation meta-gene was then used in a Cox regression model to determine probability of relapse in women with the different tumor subtypes. Differences in relapse for low (green), medium (red), and high (blue) expression are shown as tertiles in the Kaplan-Meier plots. The Kaplan-Meier curves show that proliferation adds significant survival information, beyond that gleaned from the intrinsic subtype, only for patients with Luminal A tumors (*P *= 0.012).

By applying the proliferation meta-gene to these subtypes, we show that proliferation only added prognostic information for RFS in the Luminal A subtype (Figure 4). The proliferation meta-gene was also prognostic when samples were classified more generally into 'Luminal' (Luminal A and Luminal B combined) versus 'HER2/Basal' (Additional file 1, Supplemental Figure 4), or into ER-positive versus ER-negative as clinically defined by IHC (Additional file 1, Supplemental Figure 5). It should be noted that the proliferation signature fails to further stratify ER-negative tumors (or Basal-like and HER2+/ER- tumors) because these 'groups' uniformly have high proliferation. Multivariate analyses of the NKI dataset showed that the grade, the ER status, age, and proliferation were all significant and independent predictors of survival (Additional file 2, Supplemental Tables 8 and 9).

### Co-clustering qRT-PCR and microarray data

**Figure 5:**
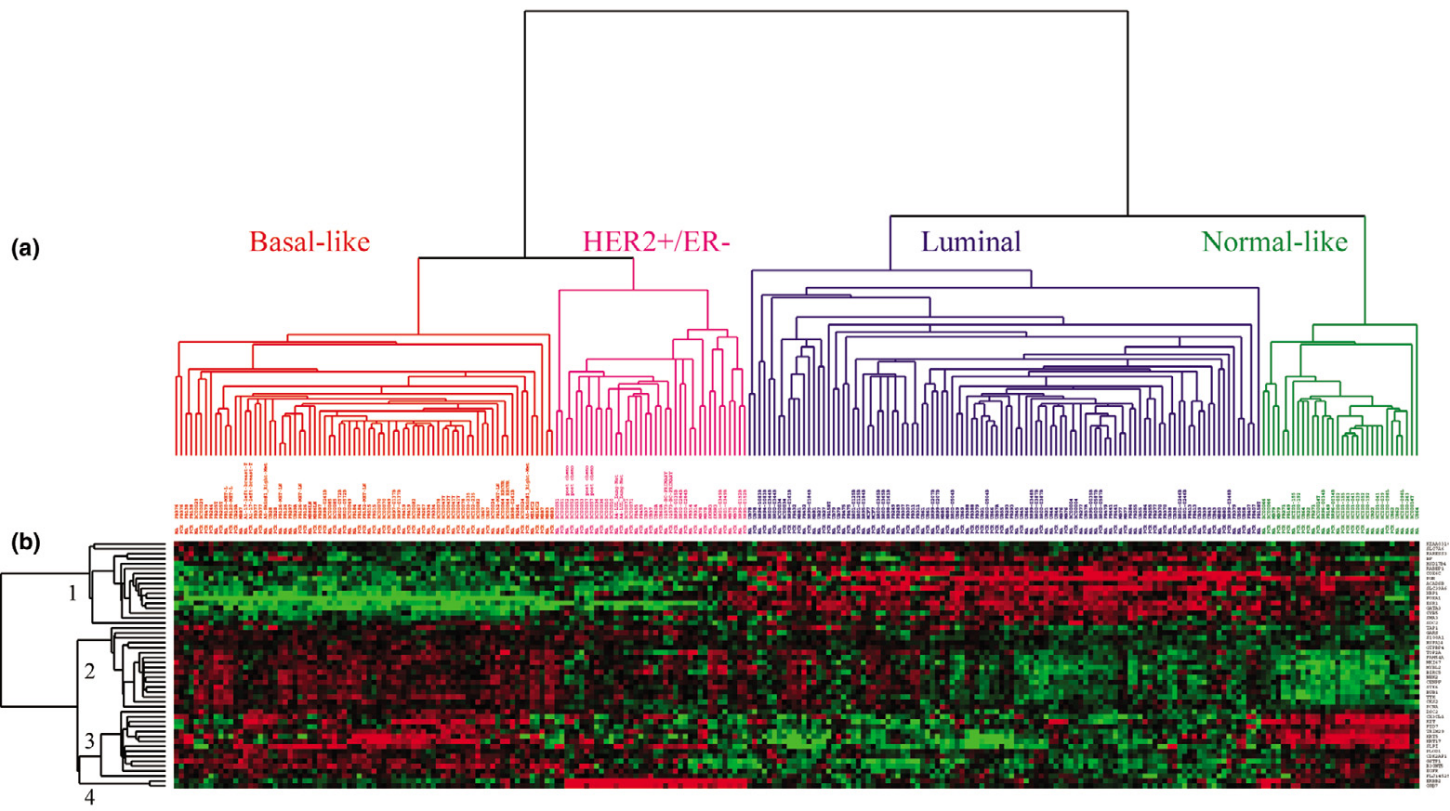
Co-clustering of real-time quantitative reverse-transcription (qRT)-PCR and microarray data using 50 genes and 252 samples. The relative copy number (qRT-PCR) and R/G ratio (microarray) for each gene was log_2_-transformed and combined into a single dataset using distance-weighted discrimination. Two-way hierarchical clustering was performed on the combined dataset using Spearman correlation and average linkage. **(a) **The sample-associated dendrogram shows the same classes as seen in Figure 1. Samples are classified as Basal-like (red), HER2+/ER- (pink), Luminal (blue), and Normal-like (green). The expression level for each gene is shown relative to the median expression of that gene across all the samples, with overexpressed genes in red and underexpressed genes in green. Genes with average expression are black. **(b) **The gene-associated dendrogram shows that the Luminal tumors and Basal-like tumors differentially express estrogen-associated genes (cluster 1); as well as basal keratins (KRT 5 and KRT 17), inflammatory response genes (CX3CL1 and SLPI), and genes in the Wnt pathway (FZD7) (cluster 3). The main distinguishers of the HER2+/ER- group are low expression of genes in cluster 1 and high expression of genes on the 17q12 amplicon (ERBB2 and GRB7) (cluster 4). The proliferation genes (cluster 2) have high expression in the estrogen receptor (ER)-negative tumors (Basal-like and HER2+/ER-) and low expression in ER-positive (Luminal) and Normal-like samples.

In order to determine whether qRT-PCR data and microarray data could be analyzed together as a single dataset, we used DWD to combine data for 50 genes across the 126 samples profiled by both qRT-PCR and microarray analyses (252 samples in total). Hierarchical clustering of these data show that 98% (124/126) of the paired samples were classified as the same intrinsic subtype and 83/126 (66%) were clustered directly adjacent to their corresponding partner (Figure 5). Microarray and real-time qRT-PCR data can therefore be combined into a seamless data set without sample segregation based on the platform. Overall, the microarray and qRT-PCR expression data showed high correlation before (0.76) and after (0.77) DWD correction.

## Conclusion

In this study we have shown that the microarray signatures for the 'intrinsic' subtype and proliferation are reproducible and prognostic using a real-time qRT-PCR assay. The biological classification by real-time qRT-PCR makes the important clinical distinction between ER-positive and ER-negative tumors and identifies additional subtypes that have prognostic value. We found that our proliferation meta-gene is a robust predictor of survival across all breast cancer patients and is particularly important for prognosis in Luminal A (ER-positive) breast cancers, which have a worse outcome than expected when proliferation is high. Work by others also supports the finding that a genomic signature of proliferation is important for predicting relapse in breast cancer, especially in ER-positive patients [28,34].

Combining microarray and qRT-PCR data provides a powerful system for discovering and then translating genomic markers into the clinical laboratory. Although these platforms are fundamentally different, the quantitative data across the methods showed a high correlation. Real-time qRT-PCR is attractive for clinical use because it is fast, reproducible, tissue-sparing, quantitative, automatable, and can be performed from archived (formalin-fixed, paraffin-embedded tissue) samples [35,36]. The benefit of using real-time qRT-PCR for cancer diagnostics is that new markers can be readily validated and implemented, making tests expandable and/or tailored to the individual. For instance, the proliferation meta-gene could be used within the context of the intrinsic subtypes or used as an ancillary test in breast cancer and other tumor types where an objective and quantitative measure of grade is important for risk stratification. As more prognostic and predictive signatures are discovered from microarray, it should be possible to build on our current biological classification and develop customized assays for each tumor subtype.

## Abbreviations

DWD = distance-weighted discrimination; ER = estrogen receptor; IHC = immunohistochemistry; PCR = polymerase chain reaction; qRT = quantitative reverse-transcription; RFS = relapse-free survival.

## Competing interests

The authors declare that they have no competing interests.

## Authors' contributions

PSB wrote the manuscript and was involved in the conception and design of the cross-platform experiments for translating the microarray 'intrinsic' classifications into qRT-PCR assays. CMP helped to write the manuscript and was involved in the conception and design of the cross-platform analyses. LP, MMu, JFQ, and NPG were responsible for conducting the qRT-PCR experiments and analyzing the molecular data. CF, JP, ZH, and XE were responsible for conducting the microarray experiments and analyzing the molecular data. AS performed the statistics for survival analyses and the correlations between microarray and qRT-PCR data. EN, MMo, HH, SSB KR, ARO, DD, RW, JPP, and OIO were responsible for patient selection and clinical data analyses at their respective institutions.

## Supplementary Material

Additional File 2 Supplemental Table 1. Clinical data for the 123-sample set prospectively analyzed by microarray and qRT-PCR Supplemental Table 2. Primer sets and gene ID Supplemental Table 3. Gene copy number relative to the reference RNA Supplemental Table 4. Forty-five paired samples for intrinsic analysis from Sorlie et al. 2003 Supplemental Table 5. Correlating classifier genes with RFS and grade using qRT-PCR assay Supplemental Table 6. Relapse free survival multivariate analyses using qRT-PCR data Supplemental Table 7. Overall survival multivariate analyses using qRT-PCR data Supplemental Table 8. Relapse free survival multivariate analyses using microarray data from NKI Supplemental Table 9. Overall survival multivariate analyses using microarray data from NKI Click here for file

Additional File 1 Supplemental Figure 1: Hierarchical clustering of Utah sample set using 'intrinsic' genes from Sorlie and colleagues (PNAS 2003). Supplemental Figure 2: Receiver Operator Curves comparing gene expression by qRT-PCR to protein expression by immunohistochemistry. Supplemental Figure 3: Kaplan-Meier plots showing survival differences between intrinsic subtypes as determined by qRT-PCR. Supplemental Figure 4: Kaplan-Meier plots showing that proliferation provides additional risk stratification only for Luminal tumors. Supplemental Figure 5: Kaplan-Meier plots showing that proliferation provides additional risk stratification only for ER-positive tumors. Click here for file

## Figures and Tables

**Table 1:** Correlating proliferation genes with relapse-free survival (RFS) and the grade using the quantitative reverse-transcription PCR assay

Gene	RFS-gene					Grade-gene	
	
	All samples	Luminal only	All samples adjusted for grade	All samples adjusted for stage	All samples adjusted for stage and grade	Spearman correlation	*P *value
BIRC5	0.136	0.0679	0.493	0.146	0.348	0.349	0.000156
BUB1	0.00247	0.0658	0.0178	0.00872	0.0258	0.45	6.84 × 10^-7^
CENPF	0.0204	0.0173	0.149	0.0934	0.295	0.469	2.00 × 10^-7^
CKS2	0.34	0.621	0.961	0.125	0.313	0.364	7.57 × 10^-5^
DUFD1	0.0525	0.0411	0.261	0.188	0.417	0.406	8.93 × 10^-6^
GTPBP4	0.000813	0.0751	0.00636	0.00177	0.00587	0.258	0.00598
HSPA14	0.0527	0.1	0.219	0.00491	0.0132	0.264	0.00467
MK167	0.101	0.0591	0.297	0.119	0.255	0.354	0.000121
MYBL2	0.00229	0.00246	0.0123	0.0318	0.0777	0.409	7.48 × 10^-6^
NEK2	0.0654	0.436	0.259	0.155	0.346	0.384	2.89 × 10^-5^
PCNA	0.155	0.166	0.179	0.182	0.152	0.151	0.109
STK6	0.000707	0.0485	0.00602	0.00816	0.0204	0.427	2.67 × 10^-6^
TOP2A	0.0347	0.291	0.142	0.0871	0.198	0.325	0.000451
TTK	0.00688	0.0651	0.0571	0.0299	0.11	0.4	1.26 × 10^-5^
Meta-gene	0.00321	0.0185	0.0252	0.0102	0.0301	0.456	4.77 × 10^-7^
