# Supplementary material for: Classification and risk stratification of invasive breast carcinomas using a real-time quantitative RT-PCR assay
Source: Breast Cancer Res. 2006 Apr 20;8(2):R23. doi: 10.1186/bcr1399 (PMC1557722; doi:10.1186/bcr1399)
Supplement: Additional File 1 — Supplemental Figure 1: Hierarchical clustering of Utah sample set using 'intrinsic' genes from Sorlie and colleagues (PNAS 2003). Supplemental Figure 2: Receiver Operator Curves comparing gene expression by qRT-PCR to protein expression by immunohistochemistry. Supplemental Figure 3: Kaplan-Meier plots showing survival differences between intrinsic subtypes as determined by qRT-PCR. Supplemental Figure 4: Kaplan-Meier plots showing that proliferation provides additional risk stratification only for Luminal tumors. Supplemental Figure 5: Kaplan-Meier plots showing that proliferation provides additional risk stratification only for ER-positive tumors. [file bcr1399-S1.doc]

**Supplemental Figures**


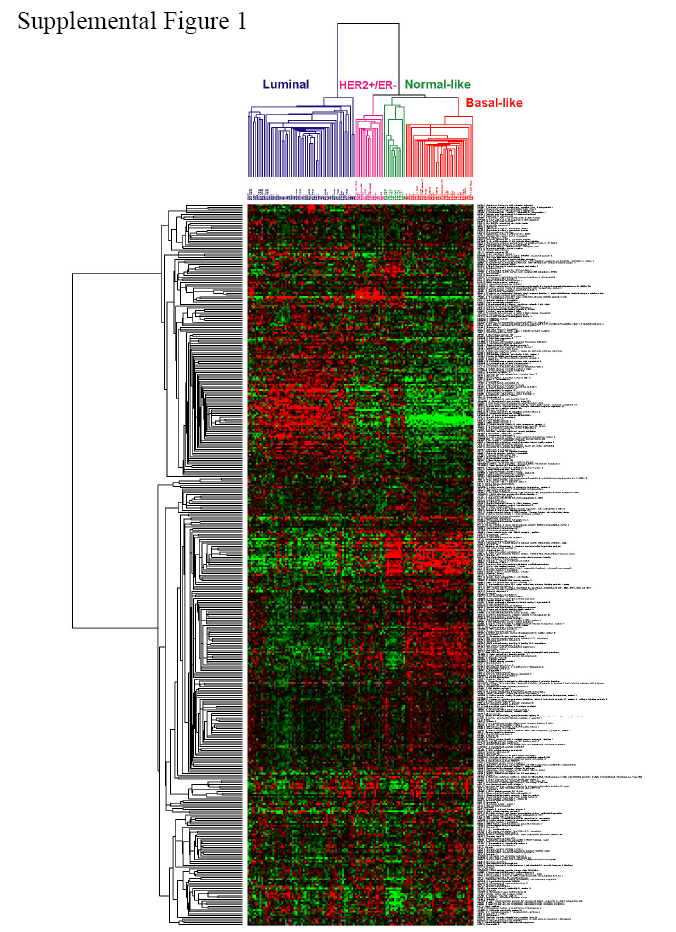


Supplemental Figure 1. Intrinsic classifications by microarray. The 402 “intrinsic” genes in common between our dataset and the data presented in Sorlie et al. 2003 were used to hierarchical cluster the 126 breast samples used in this study. The expression level for each gene is shown relative to the median expression of that gene across all the samples with high expression represented by red and low expression represented by green. Genes with median expression are black and missing values are gray. The sample-associated dendrogram shows the same classes seen by qRT-PCR (Figure 1). Samples are grouped into Luminal (blue), HER2+/ER- (pink), Normal-like (green), and Basal-like (red) subtypes.


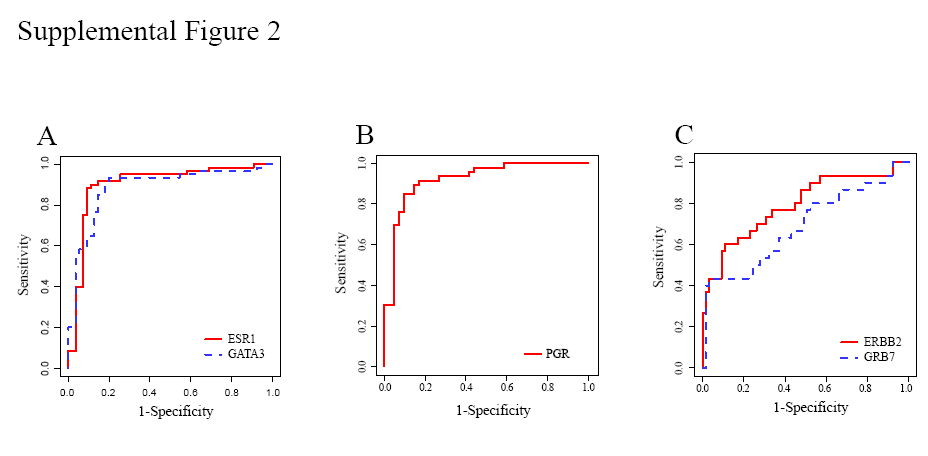


Supplemental Figure 2. Receiver Operator Curves. The agreement between immunohistochemistry (IHC) and gene expression is shown for ER (2A), PR (2B), and HER2 (2C) using ROC. A cut-off for relative gene copy number was selected by minimizing the sum of the observed false positive and false negative errors. The sensitivity and specificity of the resulting classification rule were estimated via bootstrap adjustment for optimism. Since many biomarkers having concordant expression can serve as surrogates for one another, we tested the accuracy of using GATA3 (2A-blue dotted line) and GRB7 (2C-blue dotted line) as surrogates for calling ER and HER2 protein status, respectively. We found that the gene expression of ESR1 alone had 87% sensitivity and 90% specificity for predicting ER status by IHC. The gene with the highest correlation in expression to ESR1 was GATA3 (0.79, 95% CI: 0.71 - 0.85), and GATA3 alone showed 90% sensitivity and 81% specificity in predicting ER status(2A). Gene expression of PgR correlated well with PR IHC status (sensitivity=89%, specificity=82%)(Fig 2B). There was high correlation in expression between HER2/ERBB2 and GRB7 (0.91, 95% CI: 0.87 - 0.94). Both ERBB2 (sensitivity=55%, specificity=87%) and GRB7 (sensitivity=40%, specificity=96%) had low sensitivity but high specificity in predicting HER2 status by IHC (Fig 2C).

Supplemental Figure 3. The qRT-PCR “intrinsic” subtype assay predicts survival. Kaplan-Meier curves show the distribution of time to event for relapse free survival (RFS) and overall survival (OS) in patients with Luminal tumors (blue line), HER2+/ER- (red line), and Basal-like tumors (green line). Classifications were made from real-time qRT-PCR data using the minimal 37 “intrinsic” gene list (Figure 1). Pairwise log-rank tests were used to test for equality of the hazard functions among the intrinsic classes. Patients with Luminal tumors showed significantly better outcomes for RFS (2A) and OS (2B) compared to HER2+/ER- (RFS: p=0.023; OS: p=0.003) and Basal-like (RFS: p=0.065; OS: p=0.002) tumors. This difference in outcome was significant for overall survival even after adjustment for stage (HER2+/ER-: p=0.043; Basal-like: p=0.001). There was no difference in outcome between patients with HER2+/ER- and Basal-like tumors. Tumors in the Normal Breast-like subtype were excluded from the analyses since these tumors are likely misclassified as a result of having a preponderance of normal cells in the sampling. Analyses of the same cohort using standard clinical pathological information showed that stage, tumor size, node status, and ER status were prognostic for RFS and OS (data not shown).

Supplemental Figure 4. The proliferation meta-gene adds prognostic information for the Luminal subtype of breast cancer. Tumors from the microarray breast cancer dataset were separated into 2 groups designated as HER2/Basal (combined set of HER2+/ER- and Basal-like tumors) or Luminal (combined set of Luminal A and B). The proliferation meta-gene was then applied to each group and probability of relapse was determined using a Cox regression model. The K-M curves show probability of relapse in women with Basal/HER2 (A) and Luminal (B) tumors that have low (green), medium (red), and high (blue) expression of the proliferation index. The proliferation index provides significant prognostic information to the Luminal class (p=5.7E-10), but not the Basal/HER2 class (p=0.52).

Supplemental Figure 5. The proliferation meta-gene adds prognostic information for ER-positive breast tumors. Tumors from the microarray breast cancer dataset were separated into 2 groups designated as ER-positive or ER-negative as scored by immunohistochemistry. The proliferation meta-gene was then applied to each group and probability of relapse was determined using a Cox regression model. The K-M curves show probability of relapse in women with ER-negative (A) and ER-positive (B) tumors that have low (green), medium (red), and high (blue) expression of the proliferation index. The proliferation index provides significant prognostic information to ER-positive (p=5.5E-08), but not the ER-negative class (p=0.44).
